# Exploring flexible polynomial regression as a method to align routine clinical outcomes with daily data capture through remote technologies

**DOI:** 10.1186/s12874-023-01942-4

**Published:** 2023-05-11

**Authors:** Nicole Filipow, Eleanor Main, Gizem Tanriver, Emma Raywood, Gwyneth Davies, Helen Douglas, Aidan Laverty, Sanja Stanojevic

**Affiliations:** 1 UCL Great Ormond Street Institute of Child Health, University College London, 30 Guilford Street, London, WC1N 1EH UK; 2 Great Ormond Street Hospital for Children NHS Foundation Trust, London, UK; 3 Community Health and Epidemiology, Dalhousie University, Halifax, NS Canada

**Keywords:** Remote patient monitoring, Polynomial regression, Clinical outcomes, Chronic disease, Missing data

## Abstract

**Background:**

Clinical outcomes are normally captured less frequently than data from remote technologies, leaving a disparity in volumes of data from these different sources. To align these data, flexible polynomial regression was investigated to estimate personalised trends for a continuous outcome over time.

**Methods:**

Using electronic health records, flexible polynomial regression models inclusive of a 1st up to a 4th order were calculated to predict forced expiratory volume in 1 s (FEV_1_) over time in children with cystic fibrosis. The model with the lowest AIC for each individual was selected as the best fit. The optimal parameters for using flexible polynomials were investigated by comparing the measured FEV_1_ values to the values given by the individualised polynomial.

**Results:**

There were 8,549 FEV_1_ measurements from 267 individuals. For individuals with > 15 measurements (n = 178), the polynomial predictions worked well; however, with < 15 measurements (n = 89), the polynomial models were conditional on the number of measurements and time between measurements. The method was validated using BMI in the same population of children.

**Conclusion:**

Flexible polynomials can be used to extrapolate clinical outcome measures at frequent time intervals to align with daily data captured through remote technologies.

## Introduction

Remote patient monitoring via non-invasive technologies has been widely hypothesised to reduce health care costs, increase patient autonomy, and improve clinical outcomes [1]. Technologies such as wearable devices, biosensors, and smartphones have increasingly been used in research to monitor people outside of the hospital with chronic respiratory, cardiovascular, neurological, or metabolic diseases [2]. Data captured from remote monitoring, such as heart rate and step count every second from Fitbit activity trackers, are a rich source of information on habitual metrics like physical activity that offer opportunities to explore individualised trends over time as novel predictors of clinical outcomes. The association between these novel biomarkers and clinical outcomes are important to understand to determine whether large-scale implementation of remote monitoring in chronic diseases is advantageous to patients and health care systems. However, clinical outcomes are normally captured much less frequently, leaving a substantial disparity in volumes of data from these different sources. Despite the capture of granular data from daily remote monitoring, analyses typically collapse these data into a single average measure, thereby ignoring the heterogeneity.

When there is a single, distal clinical outcome such as death, hospitalisation, or healthcare cost, a summary of the magnitude, frequency, or variability of daily data as the independent variable can be associated with the single outcome as the dependent variable [3–6] or joint-modelling can be used [7]. However, when the outcome for comparison is a continuous measure of disease severity that changes over time and is measured less frequently, the associations between daily data and clinical outcome data are more difficult to estimate. For example, researchers may want to understand the associations over time between daily habitual physical activity and nutritional status measured by body mass index (BMI), or lung function measured by forced expiratory volume in 1 s (FEV_1_), or cardiovascular health measured by blood pressure. In this case daily remote monitored data is misaligned with the clinical outcome measure (e.g., daily vs. quarterly).

Traditional methods to deal with misaligned data include collapsing data or imputation. Single imputation severely underestimates the variability of the outcome and multiple imputation is challenging to implement when the proportions of missing data are large, which is true when aligning daily data with data captured monthly or quarterly [8, 9]. Moreover, collapsing the exposure data into a small number of categories that match with the time points of outcome data is biased towards those with frequent clinical encounters, ignores the trajectory of the individual predictor, and can exclude many measurements.

The aim of this study was to investigate flexible polynomial regression as a method to estimate personalised trends for an outcome over time using FEV_1_ in children with cystic fibrosis (CF) as an example.

## Motivation

**Fig. 1:**
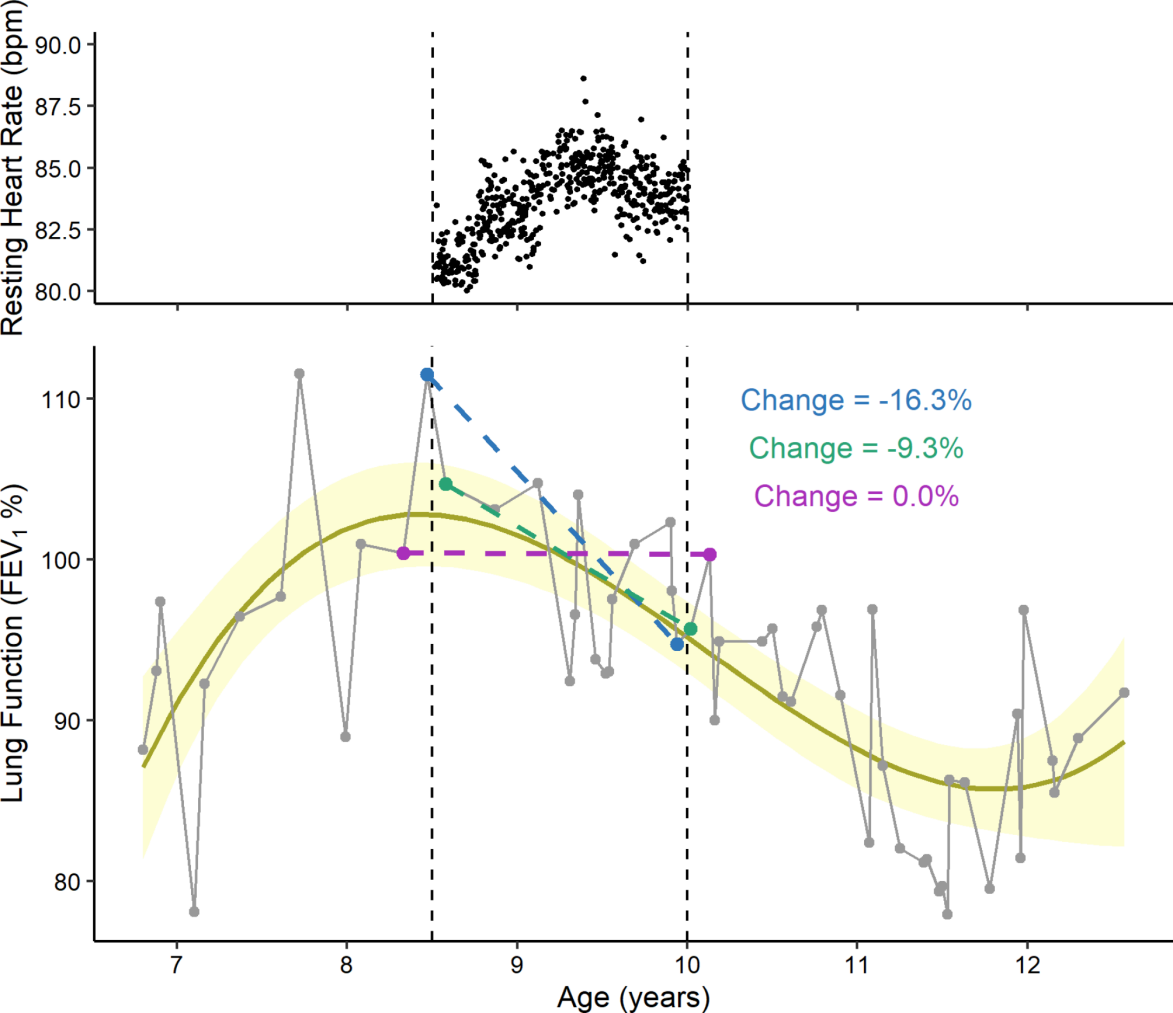
Illustration of data for an individual from a hypothetical study to demonstrate two major computational challenges with aligning asynchronous outcome measures, for example FEV_1_, with daily exposure outcomes, for example resting heart rate (dashed vertical lines represent a study window of 1.5 years; grey dots indicate measured FEV_1_ values). (1) If collapsing the 547 days of heart rate data to match the 15 FEV_1_ measurements captured during the study window, 532 (97%) days of the heart rate data would be excluded. (2) Depending on the arbitrary timepoints when baseline and end of study values are measured, the change in FEV_1_ over the study period could be a large decrease of 16.3% (blue), a small decrease of 9.3% (green), or stable with no change (purple). This has important consequences for drawing conclusions on the effects of the study, since traditionally a difference of 10–12% between measurements is used as the threshold for inferring a clinically meaningful change [10]. A proposed alternative for both issues is to use flexible polynomial regression (yellow line) to mitigate noise and/or estimate a daily, weekly, or monthly value of FEV_1_ for aligning with data captured from remote monitoring

Flexible polynomial regression is a method to predict a non-linear response variable, which is estimated by a term to an nth degree. Flexible polynomial regression was proposed as a method to estimate individualised trends in clinical outcomes to (1) align asynchronous daily data and clinical outcome data, and (2) mitigate noise when interpreting trends in outcomes (Fig. 1). The latter occurs because outcome measures including BMI, FEV_1_, or blood pressure are influenced by factors other than health status, such as patient effort, time of day, age, or treatments. This makes the interpretation of a clinically meaningful change challenging. With entire patient profiles available from electronic health records, there is opportunity for flexible polynomial regressions to estimate disease trajectories rather than relying on two points in time, e.g., at baseline and end of a study, to infer changes in health.

## Methods

### Data

Anonymised longitudinal laboratory spirometry measurements from children with CF were obtained from Great Ormond Street Hospital (GOSH) in London UK. At GOSH, the lung function laboratory measures FEV_1_ in children with CF who are old enough to complete the test (from ~4 years of age) at every outpatient clinical encounter, which is typically quarterly, as well as during hospitalisations, which are clinically indicated. A sudden drop in FEV_1_ often signals a pulmonary exacerbation, and a declining trajectory over time indicates deteriorating health. Conversely, an increase in FEV_1_ is associated with a positive response to treatment and/or an improvement in health. Although different interpretation strategies exist, traditionally a change of 10–12% is used as the threshold for inferring a clinically meaningful change between measurements [10, 11].

FEV_1_ is measured in litres (L), but it is conditional on sex, age, height, and ethnicity. Therefore, FEV_1_ was expressed as (%) predicted of a healthy population using the Global Lung Function Initiative (GLI) reference equations [12]. For simplicity, FEV_1_ % predicted is stated as FEV_1_% throughout. Individuals with FEV_1_ measurements > 1 year apart were excluded on the assumption that there was not enough data to accurately estimate fluctuations in FEV_1_ over time periods less than a year. Individuals with < 2 FEV_1_ measurements were also excluded since at least two values are required to estimate a trend.

### Analytical Plan

All analyses were carried out in R software [13]. Polynomial regression models from a 1st up to a 4th order were calculated to predict FEV_1_ over time for every individual; the model with the lowest AIC for each individual was selected as the best fit. Several restrictions were imposed. The maximum of 4 polynomials was selected to avoid overfitting. The polynomial order must also be lower than the number of values, therefore for individuals with 2 values, only a 1st order polynomial was tried, for individuals with 3 values, only a 1st and 2nd order were tried, etc. The optimal number of measurements, time between measurements, and distribution of measured values for the polynomial models to estimate individual trends in FEV_1_ were investigated by comparing the measured FEV_1_ values (observed) to the values given by the individualised polynomial (predicted).

To assess the stability of the flexible polynomial models against differing sample sizes, the method was carried out on a random sample of 80% of each individual’s measurements and the polynomial allocation was compared to the original that used all the measurements. This was carried out in 100 iterations.

### Validation

The flexible polynomial method was validated using BMI from the same population of children with CF at GOSH. As with FEV_1_, measurements > 1y apart and individuals with < 2 measurements were excluded.

## Results

### Data

A total of 8,715 FEV_1_ measurements from 291 people aged 3–18 years old between 2011 and 2021 in the GOSH lung function database were available. After excluding measurements > 1y apart and individuals with < 2 measurements there were 8,549 measurements from 267 individuals. There was an average of 32 FEV_1_ measurements per person (range: 2-150), an average of 5 measurements per person per year of data (range = 1–17), and an average of 53 days between consecutive measures (range: 1-365). Most repeated measures were within 6 months (98%). A sample of participant data and the output data from the flexible polynomial method for one individual is displayed in Table 1.

**Table 1:** Example FEV_1_ data for one individual. The 3rd order polynomial model was selected as the best fit. From the model, FEV_1_% is predicted for every day between an individual’s first and last measured FEV_1_ value. The standard error of the model is 4.58, and the r^2^ is 0.23

Model Input		Model Output
Day of Data	Measured FEV_1_%	Predicted FEV_1_%
1	67.83	67.22
2		67.24
3		67.25
4		67.27
5		67.29
6		67.31
7		67.33
8		67.35
9		67.37
10		67.39
11		67.41
12		67.43
13		67.45
14		67.47
15		67.49
16		67.51
17		67.53
18		67.55
19		67.57
20		67.60
21	67.54	67.62
22		67.64
23		67.66
24		67.69
25		67.71
26		67.73
27		67.76
28	73.21	67.78
29		67.81
30		67.83
31		67.86
32	70.68	67.88
…	…	…

### Flexible polynomial regression

**Fig. 2:**
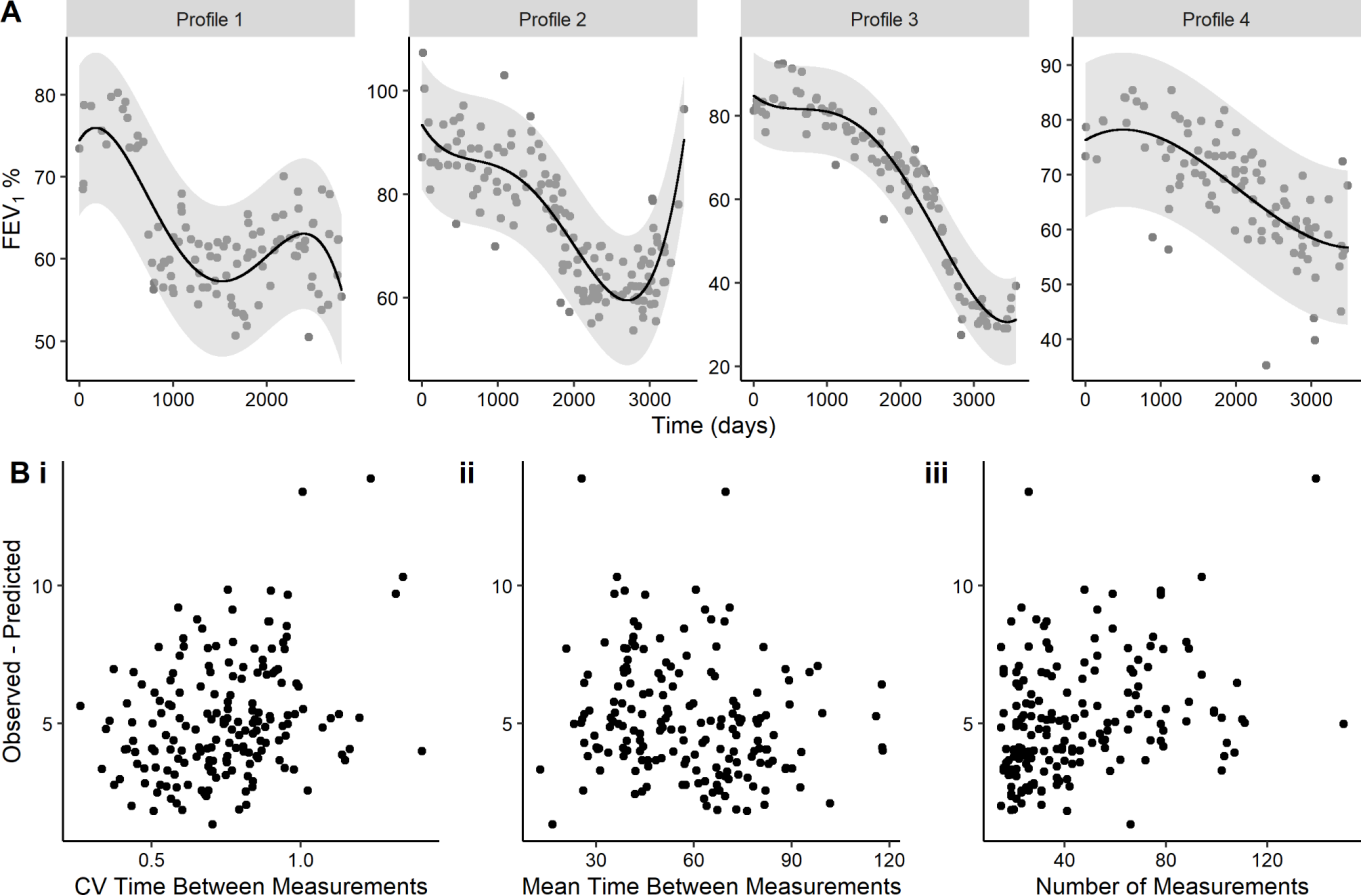
Evaluating flexible polynomials for individuals with > 15 measurements. (A) FEV_1_ profiles for four individuals with the most measurements (Profile 1, n = 150; Profile 2, n = 139; Profile 3, n = 111; Profile 4, n = 110), where grey dots indicate observed FEV_1_ measurements and black line indicates daily predicted FEV_1_ values from the flexible polynomials. These individuals have at least 8 years of data (days > 3000). (B) The observed – predicted FEV_1_ value per person compared to (i) the coefficient of variation (CV) of time between measurements, (ii) the mean time between measurements, (iii) the number of measurements

The 4th and 1st order polynomials were chosen most as the best fit (for n = 83 individuals each), followed by 3rd order for 59 individuals, and 2nd for 42 individuals. For all observations, the mean absolute difference between observed and predicted FEV_1_% was 5.5% (SD = 5.8%), which is within the range of normal variability. Nonetheless, the range was large (0.0–77.7%), thus we investigated observations where the predicted values were greater than 20% of observed values (n = 211 (2.5%)). In these instances, we observed within-person outliers of measured FEV_1_%, and not erroneous or implausible predictions (e.g., Fig. 2, Profile 4 where observed FEV_1_% < 40%).

For people with more than 15 measurements (n = 178 (67%)), the polynomial predictions worked well when individual trajectories were visualised (Fig. 2A). In this subgroup the 4th order polynomial was also chosen most often as the best fit (n = 60), followed by 1st (n = 47), 3rd (n = 42), and 2nd (n = 29). There were no significant relationships between the difference in observed and predicted FEV_1_% and the variability in time between measurements, length of time between measurements, or number of measurements, suggesting that the method is robust if there are at least 15 measurements (Fig. 2B). In CF, this equates to about 4 years of data when measurements are taken at quarterly clinics.

**Fig. 3:**
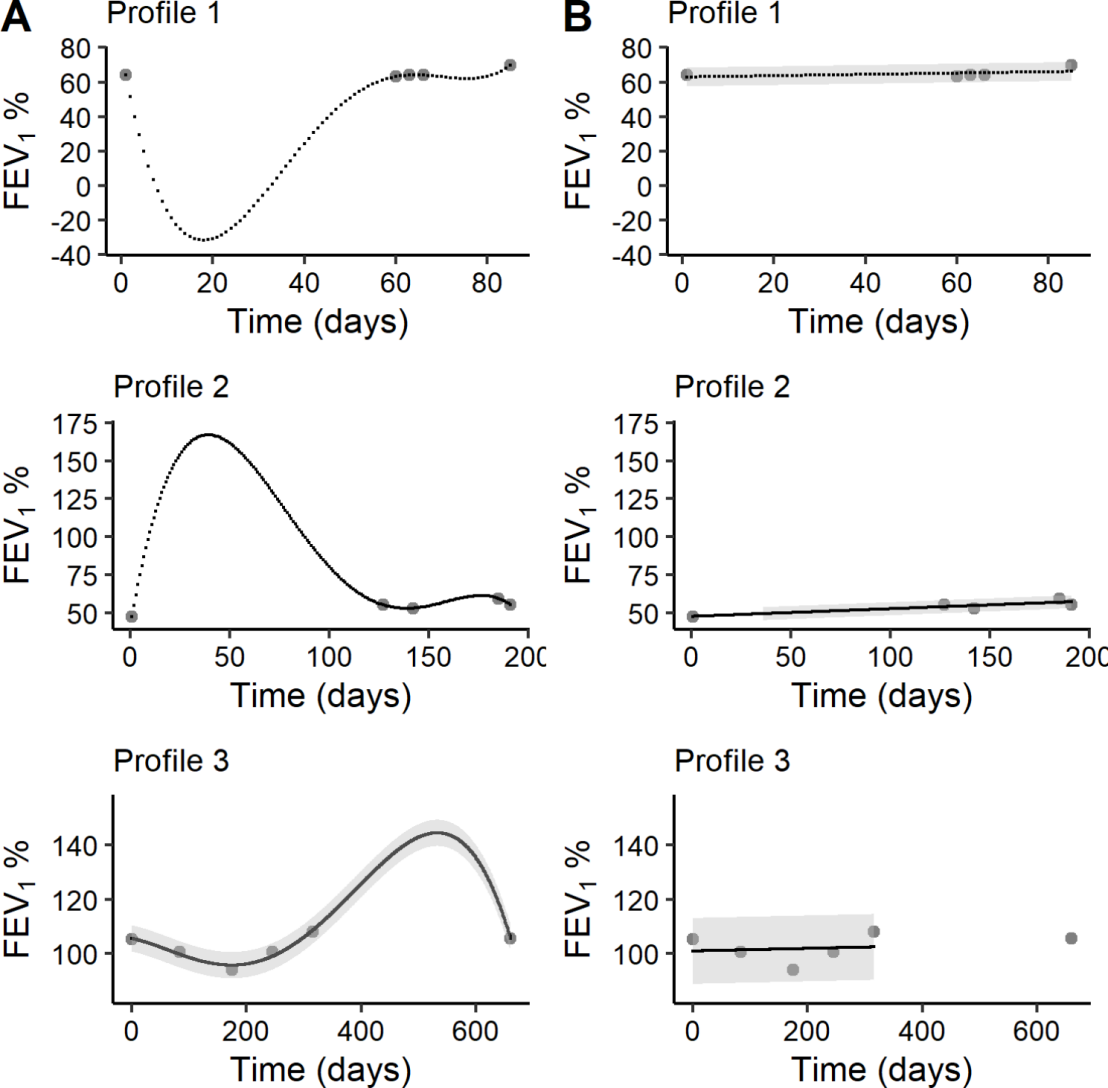
Visualisations of the flexible polynomial results across 3 example individuals with less than 8 measurements, or with 8–14 measurements but greater than 6 months between at least one observation. Points indicate observed values; black lines/points indicate daily predicted values; grey shading indicates the 95% confidence interval. (A) Flexible polynomial method with no criteria applied. Individuals with few values far apart in time had between-value predictions widely different than expected. (B) Flexible polynomial method with criteria applied. Profiles 1,2&3 have a linear model allocated because they have less than 8 measurements, and Profile 3 only has a model generated between the consecutive measurements within 6 months

When the number of measurements is sparse (i.e., < 15 measurements; n = 89 individuals), the flexible polynomial approach produces results with greater uncertainty. Investigation of individualised daily predicted values in between measured observations revealed 14 individuals that had implausible FEV_1_ values that were either negative and/or greater than 30% of the mean of the individual’s observed values (Fig. 3A). These individuals had in common 7 or fewer observations, or they had < 15 observations with a gap in time larger than 6 months between measurements, which resulted in overfitting. Rather than excluding individuals with these parameters, the polynomial models were altered to be conditional on number of measurements and length of time: those with less than 8 measurements were allocated a linear model, and those with less than 15 measurements were required to have less than 6 months between values to be included in modelling (Fig. 3B). There were 18 (14%) people with < 15 measurements but with > 6 months between at least one measurement and 60 (22%) people with < 8 measurements whom the criteria were applied to.

In this subgroup of individuals with < 15 values, the linear model was chosen most often (n = 75), followed by the 4th order (n = 9), 3rd order (n = 3), and 2nd order (n = 2). Despite forcing most individuals to fit a linear model, the mean absolute observed – predicted FEV_1_% was still low at 3.8% (range = 0.0–26.7%; SD = 3.8%), again well within the range of normal variability.

### Stability

To assess the stability of the model against sample size, the flexible polynomial method was carried out on a random sample of 80% of the measurements in 100 iterations and the polynomial assigned at each iteration was compared to the original polynomial. For over 75% of the iterations the polynomial assignment was consistent with the original. Furthermore, the majority of individuals were consistent for 100% of the iterations (n = 52). Linear models showed that the consistency of polynomial allocation within an individual was not dependent on number of observations (slope = -0.02, 95%CI = -0.16–0.12), variability of FEV_1_% (slope = -0.01, 95%CI = -0.03–0.01), mean of FEV_1_% (slope = -0.17, 95%CI = -0.27–0.00), or average time between measurements (slope = 0.07, 95%CI = -0.11–0.25).

### Extrapolation of data using flexible polynomials

The potential use of the flexible polynomial method lies in the extrapolation of an outcome value at every time point between two observed values to align the data with frequently captured exposures. To evaluate the stability of the extrapolation at different time points, the difference between all pairwise predicted values was compared to the difference between all pairwise observed values and assessed over different lengths of time for up to 6 months between measurements. This resulted in 64,573 paired FEV_1_ measurements. The mean absolute difference in predicted FEV_1_% was 0.6% (range = 0.0–24.0%, SD = 1.0%), which was much lower than the mean absolute difference in observed FEV_1_% of 6.5% (range = 0.0–81.1%, SD = 6.5%), confirming the use of the polynomial model to estimate change mitigates the noise within observed values. In a linear model the change in FEV_1_% across all pairwise comparisons were also constant at different lengths of time between measurements (slope = 1.5 × 10^− 0.5^, 95%CI = -1.8 × 10^− 0.4^ – 2.1 × 10^− 0.4^), suggesting that FEV_1_% extrapolation at 6 months is as stable as extrapolation at 1 week or 1 month.

### Validation

**Fig. 4:**
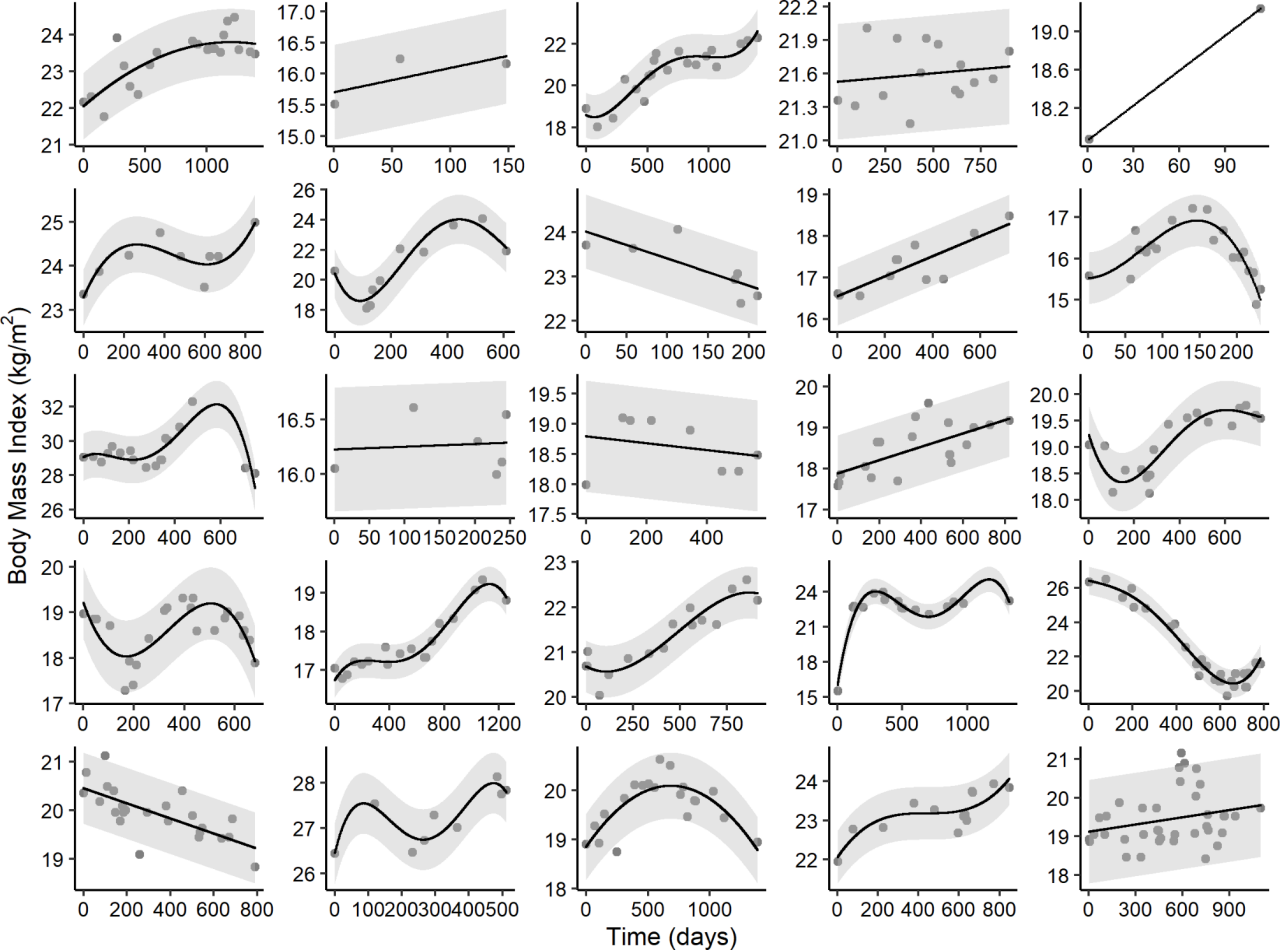
Random selection of 25 individual profiles showing flexible polynomials applied to BMI.

The method was further validated using BMI as an outcome measure in the same population of children with CF at GOSH (Fig. 4). There were 8,713 BMI measurements from 291 children 3-18y. After exclusions there were 8,547 measurements from 267 children. The 1st order polynomial was chosen most as the best fit for 87 individuals, followed by 4th order for 82 individuals, 3rd for 50 individuals, and 2nd for 35 individuals. The mean absolute difference between observed and predicted BMI was low at 0.4 kg/m^2^ (range = 0.0–7.0 kg/m^2^, SD = 0.4 kg/m^2^). These values were within the normal range of BMI variability [14].

## Discussion

The flexible polynomial model is a novel strategy to estimate individual trajectories for infrequently measured data and to align the predicted measures with data captured daily (or more frequently than the outcome). With these models a value may be extrapolated at any time point including daily, weekly, or monthly to align with data captured frequently from remote monitoring. The flexible polynomial method was developed using lung function data (FEV_1_) but also worked well for BMI from the same population and may be applicable for other continuous outcomes. Our data are from children with CF attending a single centre, but the potential application of this method extend far beyond this.

To maximise the polynomial method, entire patient profiles on outcomes are necessary. This is more feasible now with the widespread implementation of electronic health records. Moreover, it is likely that more than 8 values per person are captured within electronic health records moving forwards, especially if clinical encounters are quarterly (i.e., would require a minimum of two years of encounters). This will ensure robust use of the method as obvious errors did not occur with more than 8 values if there was a maximum of 6 months between values. Conversely, it may be appropriate to only calculate polynomial models for measurements taken during a study window to better capture the intra-individual variability over a shorter time period. Users should be careful to ensure there are enough data points over smaller lengths of time to robustly calculate individualised trends in outcomes with the method.

A potential benefit of the method is that fluctuating exposures over small time periods can be more accurately associated with outcomes. One possible example is a study investigating the effects of exercise on health. For instance, if one person exercises a small amount regularly every week, their total level of exercise over an entire study may look the same as someone who exercised a lot for 1–2 weeks, but not at all for the rest of the study. With flexible polynomial regression, the weeks of high amounts of exercise can be associated with outcomes extrapolated at the weekly level to ultimately infer a more accurate recommended level of exercise. Equally, aligning the data by exact time periods may not be optimal if you expect current exposures to influence future outcomes. For example, if someone exercises today it would likely not impact their BMI today. The binning and alignment of data to maximise the analyses should be carefully considered for each objective.

It is unclear whether the clinical outcome data should, in practice, be extrapolated at the daily level or whether daily predicted changes in outcomes are meaningful. For instance, the magnitude of an association at the daily level may not be clinically relevant since we would not expect a habitual activity to have a large daily effect. Weekly or monthly extrapolation is less speculative, but it is also unknown how accurately these extrapolated values correlate with disease severity. Extrapolation of the daily, or monthly, effect size would assume a linear association and that the exposure was constant over the period, which is not necessarily the case. Future studies should corroborate the polynomial prediction with overall trends in disease through investigation with other outcomes.

This flexible polynomial method will maximize the use of remote monitoring data to investigate detailed habitual patterns, especially where studies have not yet linked with clinical outcomes [15]. Furthermore, the potential application of flexible polynomials within longitudinally captured health data could extend beyond the ability to better align with remotely captured data; there may be non-remote data capture (e.g., continuous physiological monitoring within the intensive care setting) that may equally benefit from the method.

## Conclusion

Flexible polynomials can be used to align data captured daily via remote monitoring and infrequently measured clinical outcomes. While the method was evaluated in outcomes related to children with CF, it has a wider potential application to observational studies and clinical trials using routinely collected data, beyond CF.

## Data Availability

The data that support the findings of this study are available from Great Ormond Street Hospital, but restrictions apply to the availability of these data, which were used under license for the current study, and so are not publicly available. Data are however available from the authors upon reasonable request and with permission of Great Ormond Street Hospital.
